# Painful Diabetic Peripheral Neuropathy: Presentations, Mechanisms, and Exercise Therapy

**DOI:** 10.4172/2155-6156.S10-005

**Published:** 2013-06-30

**Authors:** Min Yoo, Neena Sharma, Mamatha Pasnoor, Patricia M Kluding

**Affiliations:** 1 Department of Physical Therapy and Rehabilitation Science, University of Kansas Medical Center, USA; 2 Department of Neurology, University of Kansas Medical Center, USA

**Keywords:** Clinical trials, Diabetic peripheral neuropathy, Painful diabetic neuropathy

## Abstract

Diabetic peripheral neuropathy (DPN) is a frequent complication of diabetes and a major cause of morbidity and increased mortality. It is typically characterized by significant deficits in tactile sensitivity, vibration sense, lower-limb proprioception, and kinesthesia. Painful diabetic neuropathy (P-DPN) is a common phenotype of DPN that affects up to one-third of the general diabetic population. P-DPN has been shown to be associated with significant reductions in overall quality of life, increased levels of anxiety and depression, sleep impairment, and greater gait variability. The purpose of this review is to examine proposed mechanisms of P-DPN, summarize current treatment regimen, and assess exercise as a potential therapy for P-PDN.

Although exercise has been shown to be an effective therapeutic modality for diabetes, its specific effects on DPN and especially the painful phenotype have not been sufficiently investigated in current literature. Several rodent models and clinical trials have presented promising results in this area, and warrant further investigations examining the effect of exercise on P-DPN.

In the United States, 18.8 million people are diagnosed with diabetes and an additional 7 million are estimated to have undiagnosed diabetes. The estimated cost of diabetes (combined direct and indirect) in the United States reached an astronomical figure of $174 billion in 2007, and continues to steadily climb, as diagnosed cases of diabetes are projected to rise to nearly 33% of all citizens by 2050 [1]. The global prevalence of diabetes mellitus in 2011 was 366 million (8.3%), and this figure is also expected to increase to 552 million (9.9%) by 2030 [2].

Diabetic peripheral neuropathy (DPN) is a frequent complication of diabetes that affects up to 50% diabetic patients in the United States [3,4]. It is a major cause of morbidity and increased mortality, and is associated with duration of diabetes, hyperlipidemia, and poor glycemic control [4]. The most common form of diabetic neuropathy is the “Distal sensorimotor polyneuropathy (DSPN)”, DPN is predominantly characterized by sensory symptoms in the “glove-and-stocking” distribution [5]. Diabetes causes DPN by promoting neuronal apoptosis and inhibiting nerve regeneration, which leads to significant deficits in tactile sensitivity, vibration sense, lower-limb proprioception, and kinesthesia [6]. Reduced or absent sensation in the foot can increase the risk of injury and wounds that may develop into serious infections requiring amputations. Diabetes can also affect autonomic nervous system causing autonomic neuropathy affecting the cardiovascular, vasomotor, sudomotor, and gastrointestinal systems [5].

## Painful Diabetic Peripheral Neuropathy

A common phenotype of DPN is painful diabetic neuropathy (P-DPN), as a fairly significant proportion of diabetic population, ranging between 10–26% depending on the sample population, experience P-DPN [7–9]. P-DPN is significantly more common in type 2 than in type 1 diabetes [9]. Pain tends to be bilateral and although it predominantly involves lower limbs, specifically the foot, in some cases, upper extremities may be involved, including fingertips and palms [10,11]. is distribution pattern occurs because the longest sensory axons are usually the first to be affected by diabetes [5]. Pain is often worse during the night, as well as under stress and fatigue [8,11]. Patients typically describe their neuropathic pain by using words such as “hot”, “burning”, “electric”, “jolts”, “sharp”, “tingling”, and “pins and needles” [11]. It may also be accompanied by allodynia (painful response to normally non-painful stimuli) and hyperalgesia (exaggerated response to mild pain stimuli) [10].

P-DPN poses a substantial and growing concern for patients and the health care system. P-DPN has been shown to be associated with significant reductions in overall quality of life in a cross-sectional study, where patients with P-DPN showed significantly poorer quality of life compared to those without neuropathy and those with non-neuropathic pain [8]. The severity of P-DPN is associated with increasing levels of anxiety and depression, as well as significant sleep impairment [12,13]. Painful neuropathy also causes considerable disability, with one-third of patients requiring a walking assist device such as a cane, walker, or wheel chair due to their neuropathy [11]. A study investigating gait function in type 2 diabetes mellitus patients with DPN found significantly greater gait variability and higher number of self-reported falls in DPN patients with painful neuropathy than in DPN patients without painful neuropathy, suggesting that pain by itself affects walking ability [14]. Although whether the neuropathic pain by itself causes significant excess mortalities in diabetes patients has not been determined, it is known that severe chronic pain is associated with increased risk of mortality [15]. Possible causes of deaths specifically attributable to pain include analgesic overdose and suicides caused by comorbid depression [16].

The exact mechanism by which diabetes causes neuropathy has not been clearly elucidated, but increased levels of advanced glycation end products (AGE) and protein kinase C (PKC) due to prolonged hyperglycemia are thought to be involved in peripheral nerve damage. Oxidative stress caused by AGE creates microscopic vascular damages, hindering blood supply to the peripheral nerves [5]. Certain pro-inflammatory cytokines including IL-6 and TNF-α are also elevated during hyperglycemia and are thought to contribute to nerve cell damage [17]. Based on these mechanisms, treating diabetic neuropathy using molecular chaperones including certain heat shock proteins (HSP) to prevent nerve injuries may be a viable option [17].

While nerve damage from hyperglycemia is a common pathophysiology shared by both the non-painful neuropathy and painful neuropathy in diabetes, painful presentations of diabetic neuropathy appear to stem from the body’s overcompensations or abnormal responses to the nerve damage. A cascade of events following the nerve damage leads to abnormal expression of sodium channels along the axon at the site of peripheral nerve damage, leading to ectopic neural discharge [18]. There is an altered expression of Na^+^, K^+^, and Ca^2+^ channels in the nociceptive neurons of the dorsal root ganglion, as well as abnormal proliferation and sprouting of sympathetic neurons. The result of these events is exaggerated or spontaneous pain sensation [18]. However, it is unclear why some people experience non-painful neuropathy while others have the painful phenotype, as painful neuropathic symptoms appear to be independent of the severity of neuropathy [19]. Hemodynamic factors have been suggested to be distinct between the non-painful versus the painful phenotypes, as epineurial intravascular oxygen saturation and blood flow were shown to be higher in people with painful neuropathy, possibly as a result of arteriovenous shunting and consequent endoneurium hypoxia stimulating painful sensation [20].

Recent studies have also implicated involvements of central pain processing mechanisms in P-DPN, showing that aberrant neurons in the ventral posterolateral thalamus of diabetes patients could become hyper-excitable and amplify the painful sensation [21]. is central process may be related to elevated thalamic perfusion [22] or abnormal supraspinal modulation of sensory processing arising potentially due to damage from prolonged hyperglycemiaf, generating allodynia and hyperalgesia [15]. Presence of a possible genetic component in P-DPN has also been suggested [11]. For instance, mutations in certain genes coding for tetrahydrobiopterin (BH4) may increase susceptibility to painful neuropathy [23].

## Diagnosis and Treatment of Painful Diabetic Neuropathy

Various methods with differing sensitivities used to diagnose diabetic neuropathy include nerve conduction test, 10-g Semmes-Weinstein monofilament examination [SWME], superficial pain sensation test, electromyography (EMG), and several variations of vibration tests. However, SWME, superficial pain sensation, and vibration testing by the on-off method for annual screening of diabetic neuropathy in diabetes and primary care clinics are important measures, with 10g monofilament test as the single most practical predictor of neuropathy [24]. Using patient’s self-report pain measures such as Numeric Pain Rating Scale, Brief Pain Inventory, Brief Pain Inventory Short Form for Diabetic Peripheral Neuropathy (BPI-DPN), and pain related subjective portion of Michigan Neuropathy Screening Instrument could provide greater knowledge about their painful symptoms. Nonetheless, P-DPN has a highly variable natural course, in which some patients spontaneously experience improvement or exacerbation, and quantifying neuropathic pain is difficult [25]. is makes delineating P-DPN from non-painful DPN a challenge and the diagnosis often primarily depends on physician’s clinical judgment and interpretation of their assessment findings. Lastly, when diagnosing P-DPN, it is important to exclude all other etiologies of neuropathy, including chronic inflammatory demyelinating polyneuropathy, B12 deficiency, hypothyroidism, and uremia [26].

Other than proper glycemic control for management of diabetes itself, the current standard care for treatment of P-DPN focuses on pharmacological treatments aiming to relieve painful symptoms. Commonly used drugs include, but are not limited to, tricyclic antidepressants (TCA), anticonvulsants (pregabalin and gabapentin), opioids, and tramadol (weak opioid agonist) [10,18,27]. All of the treatment options stated above do not alter the natural history of DPN and are only used to provide symptomatic relief via modulating pain biology, while α-lipoicacid is the only therapeutic agent targeting the etiology of DPN and currently supported by a number of studies [28]. These regimens can be effective, but they are often expensive and result in a number of adverse side effects [18]. Some medications may worsen or trigger mood disorders [27]. The dose appropriate for depression may be different from that for painful neuropathy, thus it may be difficult to administer these drugs to diabetic patients with coexisting depression. Tramadol has a potential to interact with most antidepressant medications [27]. Opioids are vulnerable to dose escalation due to rapid development of tolerance, and may cause constipation, sweating abnormalities, hypogonadism, and lowered immunity [27]. α-lipoic acidtreatment of P-DPN shows conflicting study results, and there are concerns that it may alter insulin sensitivity [27]. Furthermore, there are multiple different guidelines with conflicting information for treatment of P-DPN [27]. Reviewing recent literature examining various old and new drugs for the treatment of P-DPN reveals that while a myriad of novel drugs have been introduced with many more in clinical stages of development, finding appropriate pharmacologic therapies remains a strenuous effort [10]. Treatment of painful neuropathy continues to pose “enormous challenges” and is “currently inadequate” [18]. A group of Toronto Expert Panel on Diabetic Neuropathy, based on recent review of literature, recommended 1) TCA, 2) duloxetine (SNRI), and 3) pregabalin or gabapentin (anticonvulsants) as first-line therapies for P-DPN, and also stated a need for adequately designed studies investigating non-pharmacological approaches [28].

## Exercise Intervention in People with Painful Diabetic Neuropathy

Physical exercise and a healthy diet have been shown to improve the management of diabetes and its complications [29–31], although very few studies have investigated the effects of exercise on P-DPN. Multiple meta-analyses of randomized controlled trials [32,33] and clinical studies [34,35] suggest that exercise training of aerobic exercise, resistance training, or combined training is associated with reduction in HbA1c and improvement in functional capacity, strength, and glycemic control respectively. Exercise has also shown to have an effect on glycemic control in diabetes independent from weight control [36]. While a vast volume of literature on the effect of exercise on type 2 diabetes exists, benefits of exercise specifically on type 1 diabetes are not as clear, and evidences from available studies show that physical activity reduces insulin requirements, but may have a limited effect on glycemic control in type 1 patients [37,38].

Exercise training also improves cardiovascular complications of diabetes by ameliorating endothelial dysfunction and arterial remodeling and stiffness, likely via restoration of normal reduction-oxidation balance [39]. Treadmill exercise in diabetic mice has been shown to prevent or delay the onset of diabetic retinopathy [40]. Improvement in skeletal muscle metabolism through the enhancement of certain mitochondrial content levels is another potential benefit of regular exercise training [41]. On the other hand, the effects of exercise training on quality of life, depression, and anxiety are inconclusive at this point, as the literature has shown mixed results and may require additional well-designed randomized controlled trials [42].

Patients with DPN can safely engage in exercise. Previously, weight-bearing exercise had been contraindicated among people with DPN [43], likely due to a greater perceived risk of foot injury that may develop unnoticed by the patients. However, several prospective cohort studies found no association between increased weight-bearing activity and risk of foot ulcers [44,45]. Following this, a randomized controlled trial in 2008 showed that the intervention group who went through a self-monitored walking program and received motivational phone calls to promote weight-bearing activities did not have an increased incidence of foot ulcers compared to the control group that received only diabetes education and foot exams [43]. is has led to a recent change in exercise guidelines for people with DPN to allow moderate-intensity weight-bearing exercise [46,47].

Although exercise has been shown to be beneficial for diabetes control, its effects on DPN and especially the painful phenotype are not clear. Because poor glycemic control leading to nerve damage is thought to be one of the causes of DPN, it’s possible to conjecture that exercise is protective against DPN. Exercise may also target DPN and P-DPN by altering the microvascular components of diabetes, which is a reasonable inference from studies showing benefits of exercise on diabetic complications heavily dependent on microvascular mechanisms such as diabetic retinopathy [40]. Regular exercise can reverse reduction in nitric oxide (NO) production by the vascular endothelium in diabetes [48]. A streptozocin-rodent model study found that long-term exercise improved insulin vascular function and endothelium-dependent microvascular dilation [49]. From these evidences, we may infer that the impairment in blood supply to peripheral nerves could be prevented or even reversed by exercise. A randomized, controlled clinical trial involving diabetic patients without DPN enrolled in a prescribed and supervised 4-h/week exercise program for 4 years found that fewer participants in the exercise group developed neuropathy compared to the control group, which suggests exercise may delay or even prevent the onset of DPN in diabetic patients (Table 1) [50]. However, we cannot infer from this study whether exercise can reduce or reverse neuropathy in patients already affected by DPN. A lifestyle intervention consisting of individualized diet and exercise counseling in people with neuropathy of impaired glucose tolerance (pre-diabetic state) observed positive changes in intraepidermal nerve fiber density (IENFD)and reductions in pain according to visual analog scale (VAS) in one year (Table 1) [51].

The effects of exercise on P-DPN are largely limited to animal studies that report promising results. A mouse model study with four randomized groups (normal-sedentary, normal-exercise, STZ-sedentary, STZ-exercise) using streptozocin (STZ) to induce diabetes found that exercise training significantly decreases diabetes-associated neuropathic pain, including thermal hyperalgesia and mechanical allodynia in diabetic mice (Table 1) [17]. The investigators used standard methods of hypersensitivity assessment in animals—thermal withdrawal latency and mechanical withdrawal threshold in response to von Frey testing—to validate P-DPN. Compared to the STZ-sedentary group, the STZ-exercise group showed greater expression of Hsp72, a heat shock protein thought to protect against cell injury and repair damaged nerves [17]. Hsp72 may achieve this by acting as molecular chaperones to correct protein folding, and is elevated by skeletal muscle contraction [17,52]. A recent pilot study from our laboratory specifically exploring the effect of exercise training on DPN yielded promising data showing an increase in intraepidermal nerve fiber (IENF) branching, as well as significant reductions in pain and neuropathic symptoms in DPN patients who underwent a 10-week aerobic and resistance training program (Table 1) [53]. As a follow-up to these results, a more extensive pilot trial that focuses on aerobic exercise only while expanding outcome measures of interest and increasing specificity of certain outcome assessment methods is currently ongoing. Investigating exercise effects on neuropathic pain symptoms in clinical trials may provide insight of exercise-related benefits that are specific to P-DPN phenotype and assist in improved diagnostic tools to evaluate and treat P-DPN.

## Conclusion

P-DPN presents a tremendous challenge to the health care system as prevalence of diabetes continues to grow exponentially. Animal studies and a small number of clinical trials have shown promising improvements in pain outcomes following exercise in people with P-DPN. However, in spite of its demonstrated feasibility and potential effectiveness, exercise as a therapeutic intervention for DPN has not yet been adequately investigated. Future studies need to identify specific diagnostic criteria for P-DPN, include outcome measures that have been validated and are responsive to change, and consider the confounding influence of pharmacological management to the effect of exercise.

## Figures and Tables

**Table 1:** Studies Investigating the Effect of Exercise on DPN/P-DPN.

Author & year	Design/Methods	Results/Conclusion
Balducci et al. [50]	Randomized controlled clinical trial 4 year (4h/week) exercise program in diabetes patients without neuropathy at enrollment	Significantly more cases of sensory or motor neuropathy in the control group during the 4 years Exercise may delay or even prevent the onset of DPN in diabetic patients
Smith et al. [51]	Individualized diet and exercise counseling in patients with neuropathy associated with impaired glucose tolerance (IGT) for 1 year	Positive changes in intraepidermal nerve fiber density (IENFD) and reductions in pain in the visual analog scale (VAS) IENFD should be included as an end point in future neuropathy trials.
Chen et al. [17]	Randomized controlled trial with rodent model Daily exercise training in induced diabetic mice vs sedentary induced diabetic mice	Reductions in diabetes-associated neuropathic pain, including thermal hyperalgesia and mechanical allodynia in the exercise group Greater expression of Hsp72 in the exercise group
Kluding et al. [53]	10-week exercise and resistance training program in DPN patients	Increases in IENF branching, reductions in pain (VAS), reductions in neuropathic symptoms post exercise program
